# Control of Methicillin-Resistant *Staphylococcus aureus* Pneumonia Utilizing TLR2 Agonist Pam3CSK4

**DOI:** 10.1371/journal.pone.0149233

**Published:** 2016-03-14

**Authors:** Yi-Guo Chen, Yong Zhang, Lin-Qiang Deng, Hui Chen, Yu-Juan Zhang, Nan-Jin Zhou, Keng Yuan, Li-Zhi Yu, Zhang-Hua Xiong, Xiao-Mei Gui, Yan-Rong Yu, Xiao-Mu Wu, Wei-Ping Min

**Affiliations:** 1 Medical Laboratory, Jiangxi Provincial People’s Hospital, and Institute of Immunotherapy, Nanchang University, Nanchang, Jiangxi 330008 China; 2 Jiangxi Academy of Medical Sciences, Nanchang, Jiangxi 330006, China; 3 Jiangxi Provincial Key Laboratory of Immunotherapy, Nanchang, Jiangxi 330006, China; University Medical Center Utrecht, NETHERLANDS

## Abstract

The spread of methicillin-resistant *Staphylococcus aureus* (MRSA) is a critical health issue that has drawn greater attention to the potential use of immunotherapy. Toll-like receptor 2 (TLR2), a pattern recognition receptor, is an essential component in host innate defense system against *S*. *aureus* infection. However, little is known about the innate immune response, specifically TLR2 activation, against MRSA infection. Here, we evaluate the protective effect and the mechanism of MRSA murine pneumonia after pretreatment with Pam3CSK4, a TLR2 agonist. We found that the MRSA-pneumonia mouse model, pretreated with Pam3CSK4, had reduced bacteria and mortality in comparison to control mice. As well, lower protein and mRNA levels of TNF-α, IL-1β and IL-6 were observed in lungs and bronchus of the Pam3CSK4 pretreatment group. Conversely, expression of anti-inflammatory cytokine IL-10, but not TGF-β, increased in Pam3CSK4-pretreated mice. Our additional studies showed that CXCL-2 and CXCL1, which are necessary for neutrophil recruitment, were less evident in the Pam3CSK4-pretreated group compared to control group, whereas the expression of Fcγ receptors (FcγⅠ/Ⅲ) and complement receptors (CR1/3) increased in murine lungs. Furthermore, we found that increased survival and improved bacterial clearance were not a result of higher levels of neutrophil infiltration, but rather a result of enhanced phagocytosis and bactericidal activity of neutrophils *in vitro* and *in vivo* as well as increased robust oxidative activity and release of lactoferrin. Our cumulative findings suggest that Pam3CSK4 could be a novel immunotherapeutic candidate against MRSA pneumonia.

## Introduction

Since its discovery in the 1960s, methicillin-resistant *S*. *aureus* (MRSA) has become increasingly difficult to treat due to its resistance to commonly used antibiotics [1, 2]. The incidence of MRSA has increased and it now occurs both in healthcare settings as well as the community with community-acquired MRSA strains [2–4], thus becoming a serious public health issue worldwide. MRSA mutates have a penicillin-binding protein 2a (PBP2a), which mediates resistance to most β-lactam agents but some new generation cephalosporins and other classes of antimicrobial agents [5–7]. *S*. *aureus* has now acquired resistance to almost all antibiotics except glycopeptide and lipopeptide antibiotics, such as vancomycin and daptomycin, which are typically considered first-line agents against MRSA [8, 9]. However, it has also been reported [1, 10, 11] that MRSA strains have become less susceptible to vancomycin. The continuous increase in the prevalence of MRSA and its multi-drug resistance is a critical problem. In the past decade, studies of new treatment strategies against *S*. *aureus* infection have mainly focused on vaccines or antibodies[12]. Although several therapeutic antibodies and more than ten vaccines have been developed, none of these has been clinically approved [13–16]. Therefore, novel, alternative therapeutic approaches are in great clinical demand.

Toll-like receptors (TLR), as pattern recognition receptors, play a vital role in the detection of microbial infection and the initiation of both innate and acquired immune responses. TLR recognize pathogen-associated molecular patterns [17, 18], which are conserved between families of microorganisms[19]. Among them, TLR2, in heterodimerization with TLR1/TLR6 in humans and animals, is responsible for recognizing *S*. *aureus* and other gram-positive bacterial cell wall components [20, 21], such as lipoteichoic acid and lipoprotein[22]. Previous studies show that experimental animals, pretreated with peptidoglycan, can survive lethal doses of *S*. *aureus*, *Pseudomonas aeruginosus* and *Salmonella typhimurium* which are Gram negative bacteria [23–25]. Pretreatment with TLR2 agonist Pam3Cys, a synthetic ligand of TLR2 and TLR1, attenuated the sepsis-induced cytokine burst and protected mice from polymicrobia peritonitis [26], suggesting that ligands of TLR2 enhance immune responses against bacteria.

*S*. *aureus* is the most common cause of healthcare-associated pneumonia with high morbidity and mortality[27, 28]. However, there are few studies focusing on the beneficial or harmful immune response against MRSA infection by sensitizing TLR2 *in vivo*. In this study, we investigate the protective effect of Pam3CSK4 pretreatment on pneumonia caused by MRSA. We found that mice pretreated with Pam3CSK4 had reduced bacterial burden and mortality as well as weaker inflammatory responses. Pam3CSK4 also improved the antimicrobial activity of neutrophils. This study highlights the potential of the protective innate immune response, activated by Pam3CSK4, against MRSA pneumonia.

## Materials and Methods

### Animals and Ethics Statement

Pathogen-free Kunming (KM) mice (weight 18–20g, female) were purchased from the Experimental Animal Center, Jiangxi University of Traditional Chinese Medicine (Nanchang, China). They were kept on a 12:12 h light-dark cycle with food and water provided ad libitum. The animal experiments, approved by the Ethics Committee for Experimental Animals at Jiangxi Provincial people’s Hospital (permit number: 2014026), were carried out in strict accordance with the National Guidelines for Animal Welfare. All mice were housed in a clean room, in pathogen-free conditions, at the Institute for Animal Experimentation in Jiangxi Provincial people’s Hospital. To ameliorate animal suffering, all mice were anesthetized with 100 μl/mice 10% chloralhydrate (CHO) by intra-peritoneally before methicillin-resistant *S*. *aureus* infection. According with the principle of animal ethics, our work involve humane euthanasia, During the bacterial lethal challenge, All mice were observed at 2 h intervals for the first 48 h, and survival was monitored for at least 7 days, mice were sacrificed at the end-point or moribund (such as hunched back, ruffled fur, lethargy and inability to access food or water) by intra-peritoneally (i.p.) with 200 μl/mice 10% chloralhydrate (CHO).

### Reagents and bacteria

TLR2 agonist Pam3CSK4 was synthesized from InvivoGen (San Diego, USA). PE/CY5.5-anti- Gr-1, PE-anti-CD11b antibodies, TNF-α, IL-6, IL-10, IL-1β and TGF-β ELISA kits were purchased from BioLegend Inc. (San Diego, USA); PE/CY5.5 Rat IgG2b (κ) and PE Rat IgG2b (κ) were used as isotype controls (BioLegend Inc, San Diego, USA). methicillin-resistant *S*. *aureus* (ATCC43300) was purchased from Wenzhou Kont Biology & Technology Co. LTD (Jiangsu, China); Fluorescein isothiocyanate (FITC) was purchased from Sigma (Saint Louis, USA); Neutrophil Isolation Kit was purchased from Miltenyi Biotec (Cologne, Germany); TRIzol solution, a reverse transcription-PCR (RT-PCR) Kit and fluorescence quantitative PCR Kit were purchased from Takara Bio Inc. (Tokyo, Japan). Primers were synthesized by Invitrogen Inc. (Shanghai, China). Neutrophils Oxidative Burst Quantitative Assay Kit and ELISA Kit for Lactoferrin were purchased from Absin Inc. (Shanghai, China) and Cloud-Clone Corp. (Houston, USA), respectively.

### Preparation of *S*. *aureus* and FITC-labeled-HK-MRSA

Methicillin-resistant *S*. *aureus* (MRSA, ATCC43300) clone was cultured aerobically in tryptone soy broth (TSB) (Oxoid, Nepean, Canada) at 37°C to the midexponential phase, then washed twice and re-suspended in sterile phosphate buffer solution (PBS). FITC-labeled-HK-MRSA was prepared as follows: MRSA was collected as above; after killing at 100°C for 5 min, the bacteria was washed three times, then re-suspended in PBS. FITC was prepared at a concentration of 5 mg/ml, then added to the bacterial suspension with a final concentration of 5 μg/ml. This suspension was incubated at 37°C in the dark for 1 h followed by washing five times in PBS. Bacteria were aliquoted at 1x10^9^cfu/ml and stored in the dark at 4°C.

### Pneumonia model

**Fig 1:**
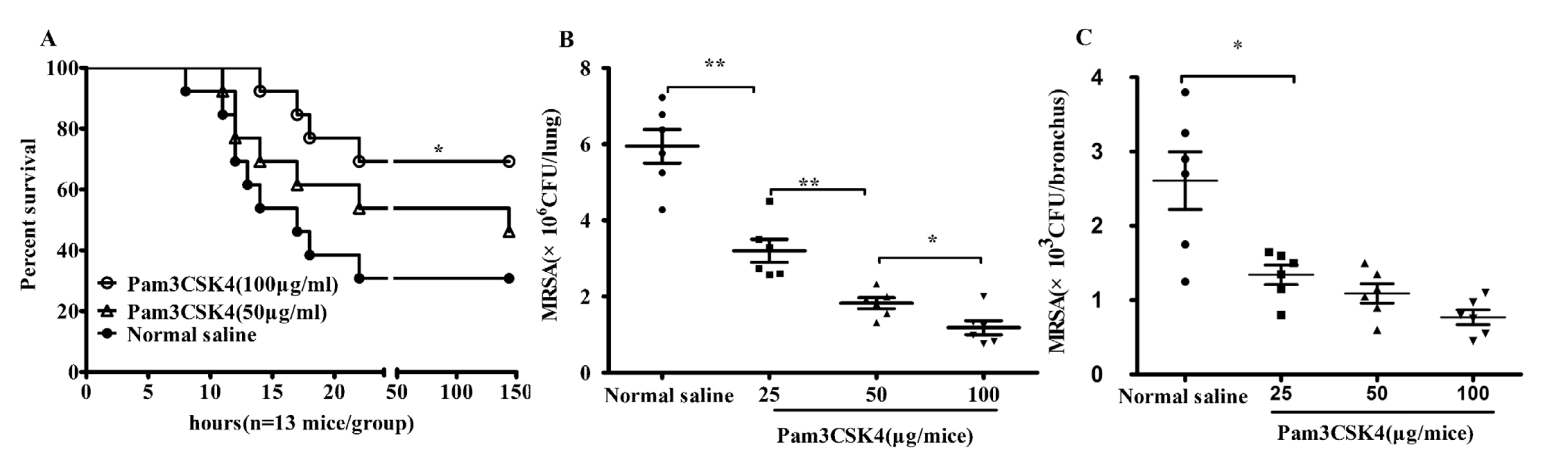
Pam3CSK4 pretreatment protects mice from MRSA infection. KM mice were anesthetized (i.p.) with 100 μl 10% chloralhydrate (CHO), then inhaled Pam3CSK4 (0, 25, 50, 100 μg /mice) in 70 μl normal saline through nasal cavity. 24 h later, mice inhaled MRSA. Survival curve of mice pretreated with Pam3CSK4 versus normal saline after intranasal infection with 1.5x10^9^ cfu MRSA(n = 13) (A). Bacterial burden in lung (B) and bronchus (C) was calculated 12 h after pulmonary infection with MRSA (2×10^8^ cfu/mice, n = 6). Note: *P<0.05; **P<0.01 *versus* normal saline-treated mice.

Female KM mice (18–20g) were anesthetized intra-peritoneally (i.p.) with 100 μl 10% chloralhydrate (CHO), then inhaled Pam3CSK4 (0, 25, 50, 100 μg/mice) through the nasal cavity. 24 h later, mice inhaled MRSA (1.5×10^9^ cfu/mice for lethal attack or 2×10^8^ cfu/mice for general attack). All mice were observed at 2 h intervals for the first 48 h, and survival was monitored for at least 7 days (Fig 1A). The whole lung and bronchus from the general-attack mice were aseptically isolated and homogenized using sterile grinders. The tissue homogenates were then diluted and plated on nutrient agar and cultured at 37°C for 18–24 h. Bacterial cfu was calculated to estimate the bacterial burden of the tissues (Fig 1B and 1C).

### Flow cytometry

**Fig 2:**
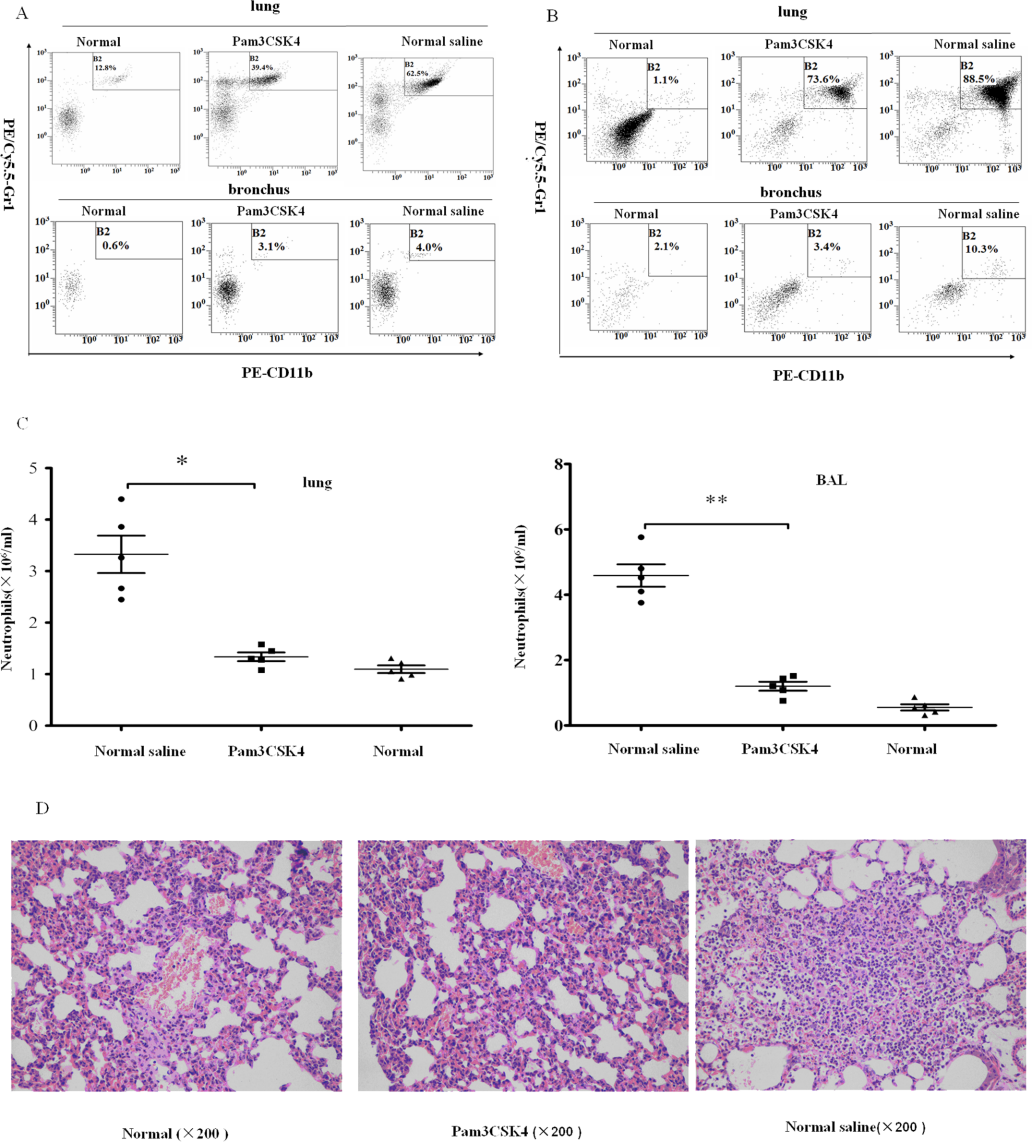
Pam3CSK4-pretreated mice have less neutrophil recruitment in MRSA-induced pneumonia. Infiltrating neutrophils were digested from lung tissues or washed out from the bronchus 6 and 12 h post infection. Cells were incubated with PE/CY5.5-anti-Gr-1, PE-anti-CD11b and analyzed by flow cytometry. Numbers of neutrophils in lung and bronchial lavage (BAL) in naïve and MRSA-challenged mice at 6 h (A) and 12 h (B) post infection were detected. The absolute numbers of neutrophils in the lungs and bronchus from mice 12 h post infection were calculated (C). Sections from MRSA-infected mice and naïve mice were stained by H&E (D). (The representative percentages of neutrophils in lung or bronchus came from one mice per group (n = 5)), *P<0.05; **P<0.01 *versus* normal saline-treated mice.

Lung neutrophilis were incubated with PE/CY5.5-anti- Gr-1, PE-anti-CD11b (BioLegend Inc.) antibodies for 30 min at 25°C in PBS containing 1% BSA (Sigma). Fluorescence was analyzed with a flow cytometer (Beckman Coulter, USA) after washing the cells with PBS three times and fixing with 4% formaldehyde(Fig 2A, 2B and 2C).

### Histopathology

At 12 h post infection, mice were euthanized, and the lungs were excised and fixed with paraformaldehyde, then embedded in paraffin, sectioned, and stained with hematoxylin and eosin (H&E) (Fig 2D).

### Enzyme-linked immunosorbent assay (ELISA)

**Fig 3:**
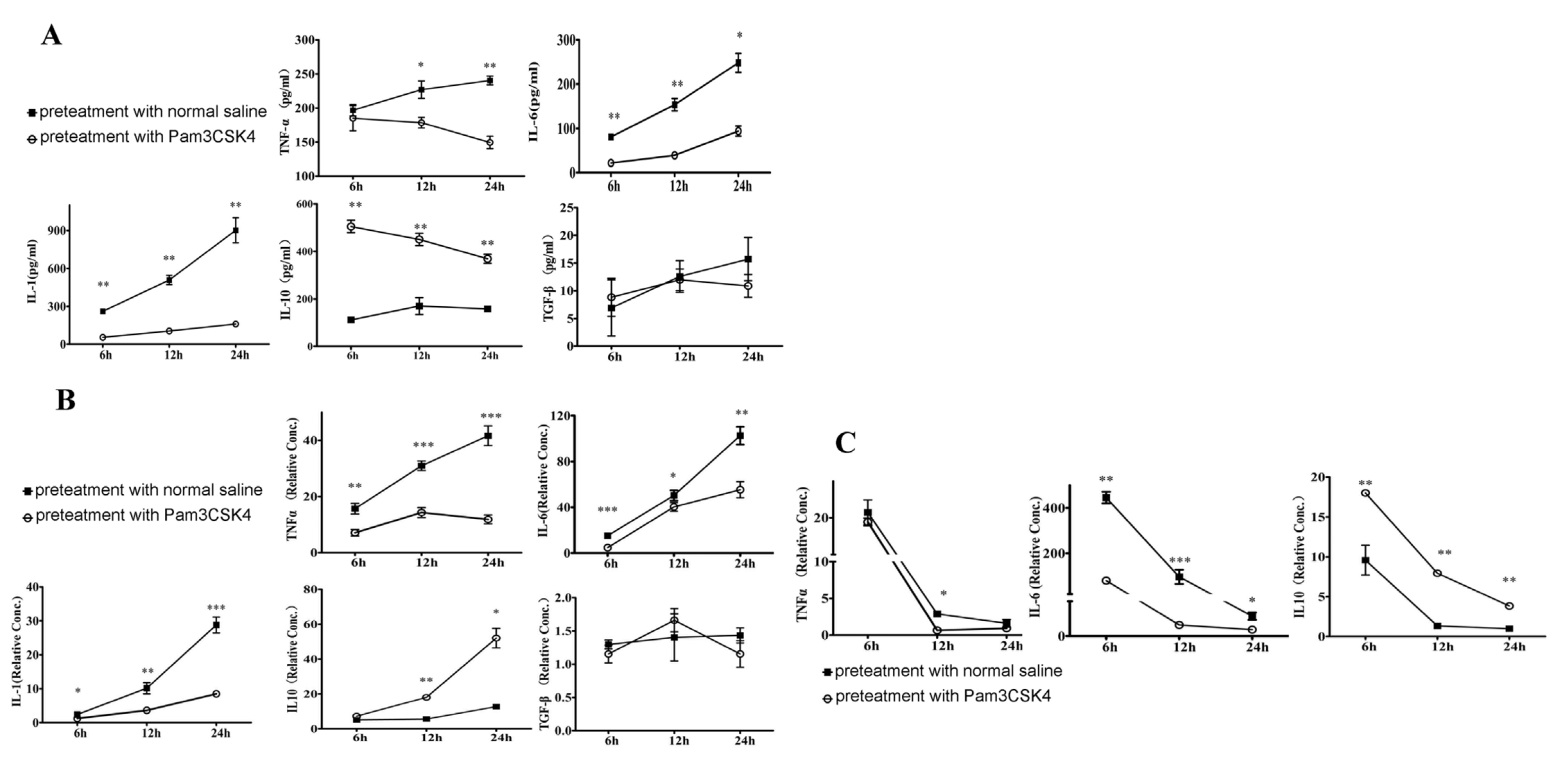
Pretreatment with Pam3CSK4 decreases inflammatory cytokine production. Lung tissues were collected and homogenized at 6, 12 and 24 h after MRSA infection. Organ extracts were centrifuged and supernatants were collected. Cytokine concentrations were measured by ELISA kits for TNF-α, IL-6, IL-1β, IL-10 and TGF-β according to the manufacturer’s instructions (A). Kinetics of cytokine mRNA levels of TNF-a, IL-6, IL-1β, IL-10 and TGF-β were tested in mouse lung at indicated times by qPCR (B). The mRNA levels of TNF-a, IL-6 and IL-10 in mouse bronchus were detected using qPCR (C). These data are representative of three independent experiments in duplicate wells (each group, n = 3–5), and the results are shown as means ± SEM. Note: *P<0.05; **P<0.01; ***P<0.001 *versus* normal saline-treated mice.

Lung tissues were collected and homogenized at 6, 12 and 24 h. Organ extracts were centrifuged (700g for 5 min at 4°C) and supernatants were collected. Cytokine concentrations were measured by ELISA kits for TNF-α, IL-6, IL-1β, IL-10 and TGF-β according to the manufacturer’s instructions (Fig 3A).

### Quantitative Reversed Transcription-PCR (qRT-PCR)

**Fig 4:**
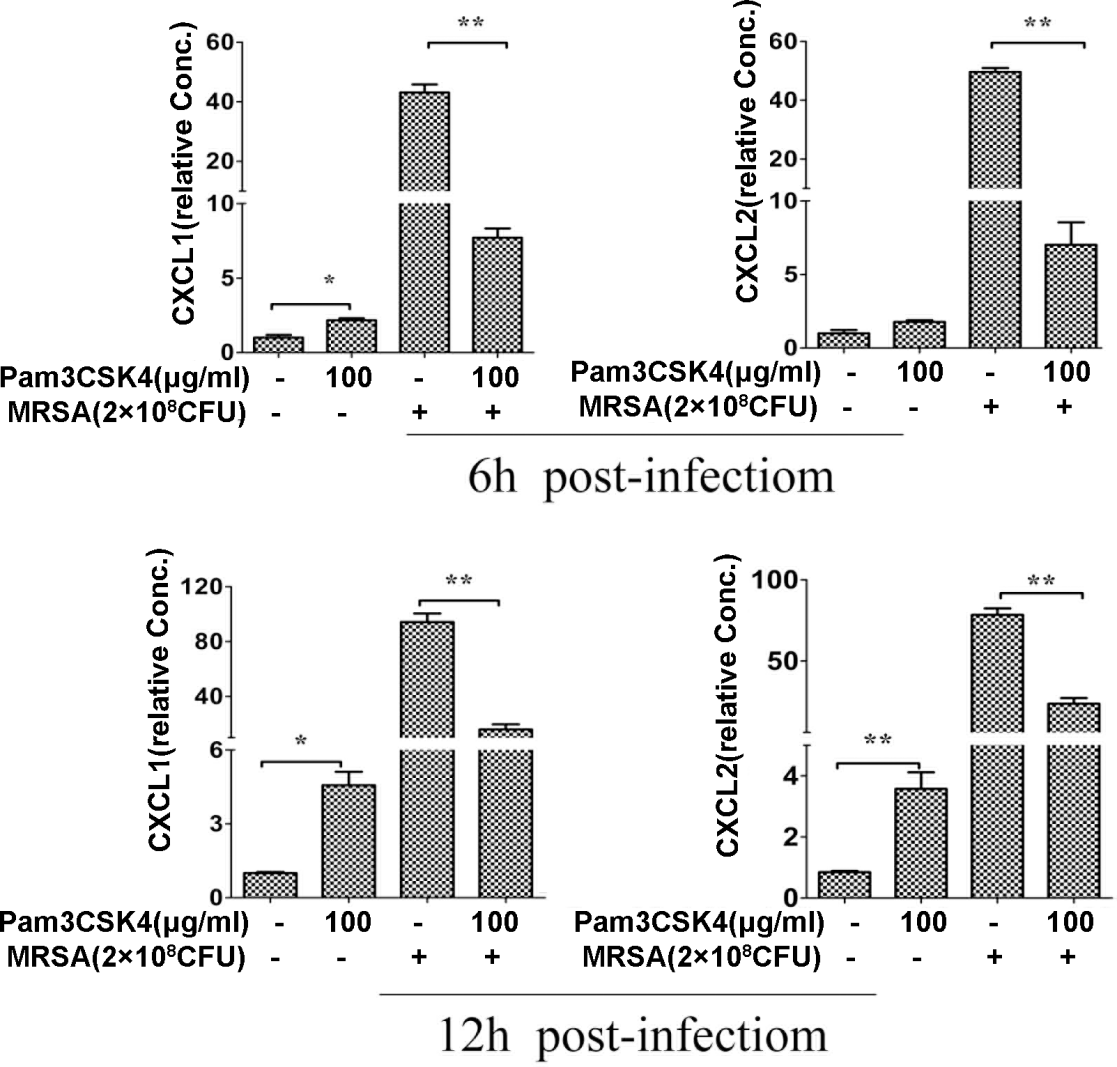
Pam3CSK4 pretreatment reduces expression of neutrophil-inducing chemokines. Lung tissues were collected and homogenized at indicated times after MRSA infection. Kinetics of cytokine mRNA levels of CXCL-1 and CXCL-2 were tested in mouse lung at 6 h and 12 h using qPCR. Data are expressed as the mean ± SEM of 3–5 mice/time from three independent experiments. Note: *P<0.05 or **p<0.01 versus saline-treated mice.

**Fig 5:**
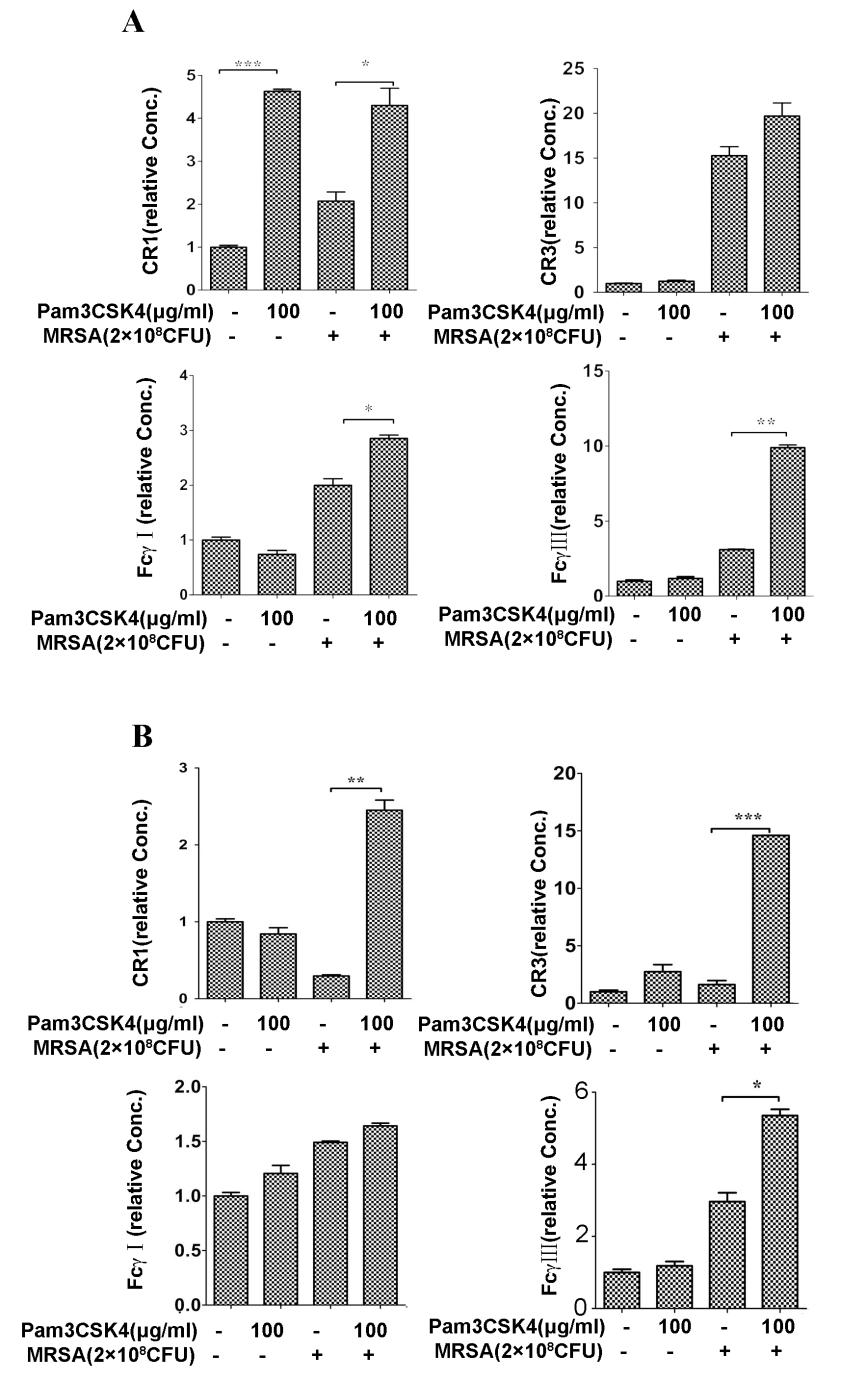
Pam3CSK4 enhances mRNA expression of Fcγ receptor (FcγR) and complement receptor (CR). KM mice were treated with Pam3CSK4 for 24 h prior to MRSA infection. After 6 h (A)and 12 h(B), the mRNA expression of FcγR(Ⅰ,Ⅲ) and CR(1,3) in lungs were performed by quantitative PCR. Data are expressed as the mean ± SEM of 3–5 mice/time from three independent experiments. Note: *P<0.05; **P<0.01 and ***P<0.001 *versus* normal saline-treated mice.

Bronchus and lung tissues were collected at 6, 12 and 24 h. Total RNA of organs was isolated using TRIzol solution (Takara) according to the manufacturer’s instructions. First-strand cDNA was synthesized from 1 μl of total RNA using a reverse transcription-PCR (RT-PCR) Kit (Takara). The cDNA was further quantitatively amplified using primers for mouse TNF-α, IL-6, IL-1β, IL-10, TGF-β (Fig 3B and 3C), CXCL1, CXCL-2 (Fig 4), FCγRⅠ, FCγRⅢ, CR-1, CR-3(Fig 5). GAPDH is used as an internal control. qPCR was performed according to the manufacturer’s instructions. Primers used in this study are listed as follows Table 1

**Table 1:** Primers used in this study.

Gene	Forward primer (5'→3')	Reverse primer (5'→3')	GenBank ID
**GADPH**	TGTGTCCGTCGTGGATCTGA	TTGCTGTTGAAGTCGCAGGA	126012538
**TNFα**	GCCAGGAGGGAGAACAGAAACT	AAGAGGCTGAGACATAGGCACC	133892368
**IL-6**	TCCAGAAACCGCTATGAAGTT	TTCATACAATCAGAATTGCCATT	13624310
**IL-1β**	CCTTGTGCAAGTGTCTGAAGC	CTCCACAGCCACAATGAGTGA	118130747
**IL-10**	GCAGCCTTGCAGAAAAGAGAG	TCCTGCATTAAGGAGTCGGTT	291575143
**TGF-β**	TATAGCAACAATTCCTGGCG	TGCTGTCACAGGAGCAGTG	6755774
**CXCL1**	ATTCACCTCAAGAACATCCAG	CTTCTTTCTCCGTTACTTGGG	229577225
**CXCL2**	CCAAGGGTTGACTTCAAGAAC	GTCAGTTAGCCTTGCCTTTGT	118130527
**FCγR**Ⅰ	CAGATGTTTCAGAATGCACAC	GAGTAGAAGAGTTCCCAGGGT	190570187
**CR3**	CCAGGA ATGCACCAAGTACA	CTCAGGATTAGCGATGCTCC	198434
**CR1**	GTCCTCTTCCTCTCCTTGCT	GAATAATAGGGTTTCCGAGC	114326525
**FCγR**Ⅲ	TGGAGATGACATGTGGCTTC	AACCATTGTGTGGAACTGTC	158508457

### Isolation and purification of neutrophils from murine bone marrow

**Fig 6:**
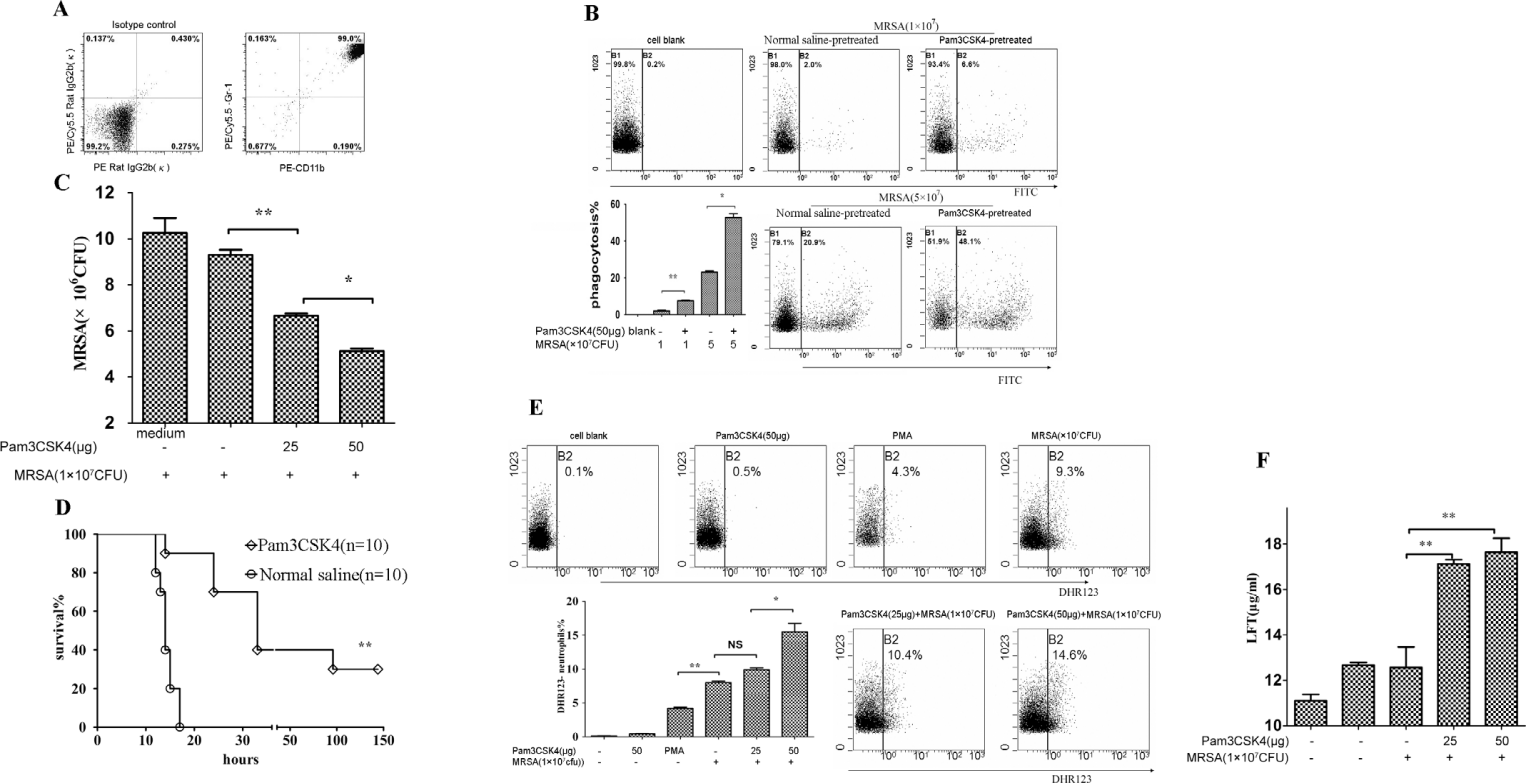
Pretreatment with Pam3CSK4 increases antimicrobial activity of neutrophils. Neutrophils were purified according to the instructions (Miltenyi,Gemany). Neutrophils were purified and measured by flow cytometry (A), and PE/CY5.5-Rat IgG2b(κ), PE-anti Rat IgG 2b(κ) as isotype controls(left panel in Fig 6A). The bacterial cfu of neutrophils was calculated (B), the left panel (the medium contol) indicated the well including MRSA and medium (RPMI1640) but Pam3CSK4 and neutrophils. FITC-positive neutrophils were quantified by flow cytometry(C). Survival of mice injected with neutrophils co-cultured with Pam3CSK4, and previously inoculated with MRSA 1 hour earlier (D). Generation of ROS was measured by flow cytometry (E) and lactoferrin release was tested using mouse Lactoferrin ELISA Kit (F). Note: *P<0.05; **P<0.01 *versus* normal saline-treated mice.

Mice were sacrificed by CO_2_ asphyxiation. The femur and tibia from both hind legs were removed, and the extreme distal tip was cut off. HBSS-EDTA (containing 0.5% bovine serum albumin and 2 mM EDTA) solution was used to wash the bone cavity with a syringe. Cells were filtered through a stainless mesh (size 70 μm), and the cell suspension was centrifuged at 300g for 10 min. Neutrophils were purified using Anti-Ly-6G MicroBead Kit (Miltenyi Biotec) according to the manufacturer's instructions (Fig 6A).

### Killing and phagocytic activity of neutrophils

Killing activity was performed as described previously [29] with a minor modification. Briefly, murine bone marrow-derived neutrophils (1×10^6^ cells/well) were cultured in a 24-well plate. Pam3CSK4 was added at final concentration 25 and 50 μg/ml at 37°C for 1 h. MRSA was added to the wells at a ratio of 10:1 (MRSA: neutrophil cells) and cultured another 2 h. Neutrophils were lysed by H_2_O (pH 11), plated on agar media in a 10-fold serial dilution, and incubated at 37°C for 18–24 h. The bacterial cfu was calculated (Fig 6B).

Phagocytosis assay was performed as described previously [30] with a minor change. Bone marrow-derived neutrophils were cultured with Pam3CSK4 (50 μg/ml) in RPMI1640 (10% fresh murine serum) for 1 h and the FITC-HK-MRSA (1×10^7^, 5×10^7^ cfu) was added into the culture for an additional hour. Cells were washed three times with PBS and FITC-positive neutrophils were quantified by flow cytometry(Fig 6C).

### Adoptive transfer of mouse neutrophils

For adoptive transfer experiments, bone marrow-derived neutrophils were co-cultured with Pam3CSK4 (40 μg/ml) in RPMI1640 (10% fresh murine serum) for 1 h. Cells (1×10^6^ cells/mice in 100 μl of sterile saline were injected via tail vein into mice that had been inoculated with MRSA through tail vein 1 h previously (Fig 6D).

### Degranulation and oxidative activity of neutrophils

Bone marrow-derived neutrophils (1×10^6^cells/well) were cultured with Pam3CSK4 (25, 50 μg/ml) in RPMI1640 (10% fresh murine serum) for 1 h, then HK-MRSA was added to the culture for another hour. Release of neutrophil reactive oxygen species (ROS) was measured using PMN Oxidative Burst Quantitative Assay Kit (Absin, Shanghai, China) according to the manufacturer’s instructions. Generation of ROS was measured by flow cytometry (Fig 6E). Lactoferrin release was tested using a mouse Lactoferrin ELISA Kit (Cloud-Clone Corp.) (Fig 6F).

### Statistical analysis

Statistical analysis of the data was performed by one-way ANOVA or the Student’s t-test using SPSS 13.0 software where appropriate. Survival data were analyzed using the log-rank test. All data are presented as mean ± SEM. The level of significance was P< 0.05. Data for flow cytometry were analyzed by CXP2.1 or FlowJo7.6 software.

## Results

### Pretreatment with Pam3CSK4 protects mice from MRSA pneumonia and increases bacterial clearance

*S*. *aureus* is the most common cause of healthcare-associated pneumonia[31, 32]. To investigate the effectiveness of Pam3CSK4 pretreatment on MRSA pneumonia, KM mice inhaled MRSA at a lethal dose 24 h after inhaling Pam3CSK4. Fig 1A shows that about 69% of mice, pretreated with Pam3CSK4 (100 μg/mice), were still alive whereas 30% in the normal saline-pretreated mice after infection (P<0.05). The Pam3CSK4-treated group had longer survival than the control group. However, mice that received a reduced dose of Pam3CSK4 pretreatment had no significant difference in survival than control mice (P>0.05). Bacterial clearance in lung and bronchus tissues was enhanced in the mice pretreated with Pam3CSK4, in comparison with mice treated with normal saline in a dose-dependent manner (Fig 1B and 1C). These results suggest that Pam3CSK4 can induce protection against lethal dose MRSA infection, and this protective ability may be associated with enhanced bacterial clearance *in vivo*.

### Pretreatment with Pam3CSK4 limits neutrophil infiltration

Neutrophils work as a critical and major cellular defense component in the innate immune system against *S*. *aureus* infection. When MRSA invades the host body, neutrophils rapidly accumulate at the infection site [33, 34]. Unfortunately, excess neutrophils can lead to acute inflammation with organ or tissue injury while working to clear MRSA [35, 36]. To investigate if Pam3CSK4 could have an impact on neutrophil infiltration, we used flow cytometry to examine the neutrophils in bronchus and lungs of mouse pneumonia models, which were identified by fluorochrome-labeled antibodies CD11b and Gr-1. Following an MRSA attack of 6 h, 39.4% CD11b^+^ Gr-1^+^ cells were detected in the Pam3CSK4-treated group, much less than the amount of cells (62.5%) in the lung control group (Fig 2A). In the bronchus, double-positive cells comprised a very small portion, yet there was still a decrease in the Pam3CSK4 treatment group (Fig 2A). After an MRSA attack of 12 h, CD11b^+^ Gr-1^+^ cells increased in both treatment and control groups. The percentage of CD11b^+^ Gr-1^+^ cells in the lung were not difference between the treatment group and the control group (Fig 2B), but the difference in bronchus between the two groups was more obvious (from 3.4% to 10.3%) (Fig 2B). The amount of neutrophils in lung and bronchus tissues of saline-pretreated mice was also more than the Pam3CSK4-treated and normal mice at12 h post-infection (Fig 2C), while there was no significant difference between Pam3CSK4-treated mice and normal mice (Fig 2C). At 12 h post infection, a large amount of inflammatory cells was observed infiltrating saline-treated mice, whereas less infiltration was seen in Pam3CSK4-pretreated and normal mice (Fig 2D) (The representative percentages of neutrophils in lung or bronchus came from one mice per group (n = 5)). These data demonstrate that pretreatment with Pam3CSK4 decreases neutrophil infiltration in lungs and bronchus of MRSA-infected mice. Neutrophils accumulated less at the infection site than the control group, implying that Pam3CSK4 is capable of repressing the host’s excessive inflammation response.

### Pretreatment with Pam3CSK4 decreases inflammatory cytokine production

Excessive inflammatory responses can be fatal for victims suffering from pathogenic infection[37, 38]. We evaluated the *in vivo* secretion of pro-inflammatory and anti-inflammatory cytokines in Pam3CSK4-treated mice. We also compared the mRNA expression of these cytokines in pneumonia mice at different post-infection times. As shown in Fig 3A and 3B, at the protein and mRNA level, mice pretreated with Pam3CSK4 express a lower level of TNF-α, IL-1β and IL-6 at 6, 12 and 24 h after lung infection. Interestingly, IL-10 transcription rapidly increased in mice pretreated with Pam3CSK4, whereas the expression of TGF-β was no different between Pam3CSK4 pretreatment group and the control group. Next, we detected the expression of pro-inflammatory and anti-inflammatory cytokines in bronchus tissues and found that the expression of IL-1β, IL-6 and IL-10 was similar to that in lung tissues (Fig 3C). These results indicate that Pam3CSK4 pretreatment decreases the inflammatory response by reducing the expression of inflammatory cytokines and increasing the level of anti-inflammatory cytokines.

### Pretreatment with Pam3CSK4 decreases chemokine expression

CXCL-2 and CXCL1 are chemoattractive for neutrophils as they recruit them to the site of infection [39]. To identify the effect of Pam3CSK4 pretreatment on CXCL-2 and CXCL1, we investigated their expression using qPCR (Fig 4). Both CXCL1 and CXCL-2 expression decreased in Pam3CSK4-pretreated mice at 6 and 12 h post infection (Fig 4). Interestingly, Pam3CSK4 pretreatment alone (without MRSA infection) slightly elevated CXCL1 and CXCL-2 expression. The results suggest that Pam3CSK4 inhibits chemokine expression after MRSA infection, thus repressing excessive neutrophil infiltration and subsequent inflammatory response.

### Pretreatment with Pam3CSK4 increases expression of Fcγ receptors and complement receptors

Fcγ receptors (FcγR) are opsonin-dependent receptors employed by phagocytic cells to recognize microorganisms. FcγR are divided generally into three main classes: high-affinity receptor for monomeric IgG: FcγRI and low affinity for monomeric IgG, FcγRII, and FcγRIII [40].

Complement receptors (CR) are another opsonin-dependent receptor. CR includes CR1 and CR3, which bind to the C3b and iC3b fragments of the C3 component of complement, respectively [41]. To analyze the impact of Pam3CSK4 on the phagocyte surface of these receptors, we used qPCR to estimate their expression in lungs of pneumonia models at 6 h and 12 h after MRSA infection. The levels of CR1 and FcRⅢ in the Pam3CSK4-treated group exceeded the control group at both timepoints. Expression of CR3 in lungs was the same between Pam3CSK4-preteated mice and saline-treteated mice at the first 6 h post infection, although saline-preteated group decreased significantly than the Pam3CSK4- pretreated group in additional 6 hours post infection. FCRⅠexpression did not become evident at 12 h even though there was a small increase in the Pam3CSK4-pretreated mice at the first 6 h post infection (Fig 5). The results demonstrate that Pam3CSK4 can stimulate expression of phagocytosis-relative receptors.

### Pretreatment with Pam3CSK4 increases antimicrobial activity of neutrophils

Neutrophils are the main phagocytic cell in the blood and the first line of defense against bacterial infections [33, 34]. Here, we assessed the killing and phagocytic activity of bone marrow-derived neutrophils after Pam3CSK4 treatment *in vitro* and *in vivo*. Bone marrow-derived neutrophils were isolated and identified (Fig 6A). Pam3CSK4-treated neutrophils showed stronger bactericidal or bacteriostatic activity than the control group in a dose-dependent manner (Fig 6B). In addition, we performed a phagocytosis assay. Phagocytic activity in Pam3CSK4-treated neutrophils was significantly enhanced in comparison to the control group (Fig 6C).

Next, we tested the protection of Pam3CSK4-activated neutrophils on mice chanllenging by MRSA via adoptive transfer experiments *in vivo*. Our results showed that survival of mice pretreated with -activated neutrophils was notably longer than that of mice pretreated with saline (control group) (Fig 6D).

Finally, we assessed the degranulation and oxidative activity of neutrophils. The results demonstrate that Pam3CSK4-treated neutrophils had stronger oxidative activity and released more lactoferrin than the control group (Fig 6E and 6F). These findings suggest that Pam3CSK4 can improve neutrophils’ antimicrobial activity by enhancing their oxidative activity and phagocytic ability.

## Discussion

Methicillin-resistant *S*. *aureus*, which can cause severe pulmonary and blood infections with high morbidity and mortality, is now drawing greater attention than ever before. A limited variety of antibiotics have been used to control the spread of MRSA, but some of these are no longer effective. In recent years, new vaccines and antibodies have been developed [42–48]; however, none of these has been clinically approved [13–16]. Despite a large number of anti-microbial choices and supportive care, these therapeutic strategies have failed to reduce mortality in severely septic patients, partly due to the inflammatory response associated with excessive pathogens [37, 38, 49, 50]. It is essential for the host to control moderate inflammatory responses during infection, and the release of pro-inflammatory and anti-inflammatory cytokines acts as an early stage marker and prediction of mortality from sepsis [51–53].

It is evident that novel, alternative therapeutic approaches must be developed. Some innate immune regulators have been used to trigger and modulate the host systemic antibacterial response, for example, IDR-1, an innate defense-regulator peptide, protected mouse models from infections including *S*. *aureus*[54]. TLR2, responsible for recognizing the gram-positive bacteria *S*. *aureus*, possesses a protective function during *S*. *aureus* infection by regulating inflammatory cytokine responses [20, 21]. These findings led us to question whether innate immune enhancement with Pam3CSK4, a TLR2 agonist, could be exploited to reduce the impact of MRSA infection. In this study, our results demonstrate that intranasal administration of Pam3CSK4 reduced the bacterial burden and mortality in murine models with MRSA pneumonia.

Interestingly, in our present studies, we found that mRNA and protein levels of pro-inflammatory TNF-α, IL-1β and IL-6 decreased in the bronchus and lung of mice pretreated with Pam3CSK4 at early-stage MRSA infection. The anti-inflammatory IL-10, but not TGF–β, increased in Pam3CSK4-pretreated mice. Thus, Pam3CSK4 pretreatment offers protection against lethal pneumonia induced by live MRSA as a result of enhanced bacterial clearance and limited inflammatory responses.

Neutrophils are the main and first cell populations to infiltrate into lesional sites and other organs to fight infections. Unfortunately, the increased counts of neutrophils may contribute to, not only local damage of lesional sites, but also secondary damage to unaffected bystander organs or tissues[35, 36, 55], thereby promoting the development of multiple organ failure. On the other hand, it has been shown that neutrophil-depleted mice have reduced bacterial clearance ability and poorer survival rates[56]. Consistent with a previous study [57], our results show that pretreatment with Pam3CSK4 reduced, but did not deplete, neutrophil infiltration in lungs and bronchus of mice with MRSA pneumonia. This phenomenon was related to the lower expression of CXCL-2 and CXCL1, which are necessary for neutrophil recruitment in the lung[39]. Thus, Pam3CSK4 pretreatment can limit the host’s excessive inflammatory response by reducing infiltration of neutrophils.

Fcγ receptors (FcγR), an opsonin-dependent receptor on phagocytic cells, bind to Immunoglobulin G (IgG) in serum, resulting in phagocytosis and inflammatory cytokine production [40]. Human peripheral blood monocytes, treated with the TLR2 agonist, showed significantly enhanced FcγR-mediated cytokine production as well as phagocytic ability *in vitro*[58]. Consistently, our results show that 12 hours after MRSA infection, the mRNA level of both FcγRⅠand FCγRⅢ in lungs increased in mice pretreated with Pam3CSK4. However, there was not a significant increase in mice treated with Pam3CSK4 alone, and the underlying mechanism remains unclear. Another phagocytosis-relative receptor, complement receptor (CR), that binds the C3b and iC3b fragments of the C3 component in serum could lead to bacteria nonopsonic phagocytosis. Its expression increases in patients suffering from bacterial infection[59]. In this study, we found that Pam3CSK4 pretreatment increases mRNA expression of CR1 and CR3 in murine lungs challenged by sub-lethal doses of MRSA. These results suggest that the decrease of bacterial burden and mortality in mice pretreated with Pam3CSK4 is probably associated with the increasing expression of phagocytosis-relative receptors.

In the early stages of sepsis, neutrophils play an essential role in the host defense against bacterial invasion and growth. They are recruited by the host to prevent further development of the infection [60]. In this study, we found that mice with Pam3CSK4 pretreatment had better bacterial clearance ability and improved survival rates with less neutrophil infiltration in the lung. This suggests that Pam3CSK4 can probably enhance neutrophil functions. Current studies show that, with Pam3CSK4 pretreatment, neutrophils have stronger bactericidal activity and phagocytic activity, and administration of activated neutrophils helped mice survive the challenge of MRSA. Robust oxidative activity and release of lactoferrin are essential features of neutrophil activation during bacterial infection [61]. Further studies show that Pam3CSK4 itself cannot activate bone marrow-derived neutrophils to strengthen oxidative activity and release lactoferrin; however, it can activate cooperation between bone marrow-derived neutrophils and peripheral neutrophils during MRSA infection although the underlying mechanism is unclear.

## Conclusion

Our results demonstrate that mice undergoing intranasal administration of Pam3CSK4 had less bacterial burden, lower mortality, and weaker inflammatory responses to MRSA. Pam3CSK4 can improve the antimicrobial activity of neutrophils by activating cooperation between bone marrow-derived neutrophils and peripheral neutrophils. These results suggest that Pam3CSK4 could be a potential novel immunotherapy candidate against MRSA.

## Abbreviations

MRSA: methicillin-resistant *S*.*aureus*
S.aureus: Staphylococcus aureus
CFU: colony-forming units
TLR: Toll like receptor
HK-MRSA: heat killed MRSA.
